# Accuracy of automated analysis in cephalometry

**DOI:** 10.1016/j.jds.2024.09.012

**Published:** 2024-10-08

**Authors:** Réka Bagdy-Bálint, Gergely Szabó, Örkény H. Zováthi, Bendegúz H. Zováthi, Ábris Somorjai, Csenge Köpenczei, Noémi Katinka Rózsa

**Affiliations:** a Semmelweis University, Department of Paediatric Dentistry and Orthodontics, Budapest, Hungary; b Pázmány Péter Catholic University, Budapest, Hungary; c Ceph Assistant Ltd., Budapest, Hungary

**Keywords:** Artificial intelligence, Automation, Cephalometry, Clinical decision-making, Diagnosis, Computer-assisted

## Abstract

**Background/purpose:**

Artificial intelligence (AI) has been widely used in medicine, including orthodontics. The aim of this study was to investigate the training process of a cascaded Convolutional Neural Network (CNN), built for landmark detection on various qualities of lateral cephalograms and to determine the speed, reliability and clinical accuracy of an algorithm for orthodontic diagnosis.

**Materials and methods:**

The CNN model was trained on a total of 1600 lateral cephalograms. After each training datasets (input of 400, 800, 1200, 1600 images) were added, the model was evaluated on a test set containing 78 images of varying quality. We measured the accuracy of AI-based landmark detection by statistical analysis of intra- and interexaminer distance errors, as well as examiner versus model predictions, furthermore by prognosis of consecutive diagnostic failures.

**Results:**

There was a clear improvement in time efficiency (5.25 min), and substantial improvements were observed during the training process. In terms of accuracy, based on Euclidean distance error measurements, the best model provided more consistent dot tracing than two different examiners or the same examiner on two different occasions. Angular (0.05°–1.86°) and proportional (3.14%) errors, measured by the best model, were considered clinically acceptable.

**Conclusion:**

The application of a proper AI-algorithm for orthodontic cephalometric analysis results in lower variability between models than the variability observed among experts. AI predictions supported the examiners in finding the correct location of the specific landmarks more accurately and in less time as the training of the automatic prediction model improved. Further research could investigate the therapeutic consequences.

## Introduction

Machine Learning (ML) plays a crucial role in a wide range of modern professions, including medicine and orthodontics.^1^^,^^2^ Technological innovations are the driving forces behind the rapid advancement of modern dentistry, as reflected in studies of the last decades, focusing on the use of Artificial Intelligence (AI) tools to optimize particular diagnostic workflows.^1^^,^^3^

Numerous reviews show promising results in the application of AI in the early prediction of treatment needs, in determining the demand for orthognathic surgery or tooth extraction, in predicting cephalometric landmarks on 2D or 3D radiographs, as well as in identifying maturational properties of a growing patient.^2^, ^3^, ^4^ The reliability of these AI-assisted software applications is influenced by several factors; beside others, by the quality of the input dataset, the number of training cycles of the algorithm, and the characteristics of the algorithm itself. According to the latest cephalometric study in Journal of Dental Sciences, shared by Lee et al.,^4^ the standardization of manual landmark detection, image quality, and image sample might affect tool performance. Kim et al.^5^ highlight that the variability of errors in these models built for automated cephalometric analysis goes beyond individual landmarks, algorithms, or training image quantities, but also identifies inconsistencies across institutional outcomes.^5^

Statistical analysis of AI-assisted cephalometric evaluations have been conducted using various algorithms trained on diverse quantities of training sets.^5^, ^6^, ^7^ Some studies show favorable outcomes even with limited data (n = 1028),^6^ and others achieve comparable results with larger input datasets (e.g. n = 1792 or n = 3150).^5^^,^^7^ According to Kang et al.,^8^ the mean distance error in determining cephalometric landmarks by different AI-algorithms ranges from 1.1 to 4.09 mm, based on findings from 3 reviews, summarising more than 165 studies on the topic.^8^ Distance errors between manually annotated and model-predicted landmark coordinates are typically defined as Euclidean distances.^5^^,^^9^ Assessing the clinical relevance of landmark detection accuracy in cephalometric analysis is challenging as subjective diagnostic estimations of clinicians often contain meaningful errors, even with repeated evaluations of the same examiner. Ana R. Durao et al.^10^ have revealed that despite a considerable number of publications on cephalometric analysis (n = 968), only a limited number of studies have examined the validity and reliability (n = 16) of 2D landmark detection on cephalograms, as articles prioritise 3D assessments.^10^ The integration of AI prediction models into everyday processes has substantially altered this landscape over the past decade.^11^, ^12^, ^13^ However, there are still insufficient data on diagnostically and therapeutically relevant metrics measured by various tools for cephalometric analysis.

Most studies examine a maximum of 20 landmarks and disregard difficult-to-detect profiles and tangent points that are used by specialists in clinical practice but can degrade statistics. This study considers 48 cephalometric landmarks (Table 1) and compares evaluations of images of varying quality, using models trained on four datasets of different sizes. The evaluation with these landmarks covers dental, dentoalveolar- and alveolar deviations examined, based on the Rickett's and Hasund analysis, and can be used to analyse the entire skull, jaw relationships, dentition and profile.

**Table 1:** Names and abbreviations of the cephalometric landmarks detected on all digital radiographic images.

Number	Name	Abbreviation
	Calibration point 1	Cal 1
	Calibration point 2	Cal 2
1.	Mesial apex of mandibular 6	1LoMma
2.	Mesial apex of maxillary 6	1UpMma
3.	Downs A-point	A
4.	Articulare	Ar
5.	Downs B-point	B
6.	Basion	Ba
7.	Columella	Co
8.	Condylion	Cond
9.	Center of symphysis	D
10.	Soft tissue glabella	Gl'
11.	Gnathion	Gn
12.	Soft tissue gnathion	Gn'
13.	Infradentale	Id
14.	Mandibular notch point	Im
15.	Lower incisor apex	La
16.	Lower incisor crown tip	Li
17.	Lower lip anterior point	Ll
18.	Upper incisor labial outline	Ls1u
19.	Mesial cusp of maxillary 6	M6lo
20.	Menton	Me
21.	Soft tissue menton	Me'
22.	Nasion	N
23.	Soft tissue nasion	N'
24.	Orbitale	Or
25.	Supra pogonion	PM
26.	Pronasale	Pn
27.	Porion	Po
28.	Pogonion	PoG
29.	Prosthion	Pr
30.	Pterygoid point	Pt
31.	Sella turcica midpoint	S
32.	Center of sella's entry	Se
33.	Submentale	Sm
34.	Subnasale	Sn
35.	Posterior spine nasal	SnA
36.	Anterior spine nasal	SnP
37.	Stomion inferius	Stm-i
38.	Stomion superius	Stm-s
39.	Tangent 1/Gonion posterior	T1
40.	Tangent 2/Gonion Inferior	T2
41.	Trichion	Tr
42.	Mesial cusp of maxillary 6	U6
43.	Distal contact of maxillary 6	U6d
44.	Upper incisor apex	Ua
45.	Upper Incisor crown tip	Ui
46.	Upper lip anterior point	Ul
47.	Condylion posterior	ppCond
48.	Soft tissue pogonion	sPoG

Among the experiments with available software solutions, the model we tested is notable for its comprehensive, criteria-based assessments that directly investigate the physician-AI relationship. To minimize clinician-induced errors in landmark detection accuracy, measurements were repeated eight times; twice by two independent experts on four separate occasions.

In this study, our aim was to prove the significance of the quality and quantity of training data for the accuracy and the time efficiency of a ML model (hereafter‘AI’) in clinical applications, using a relatively large dataset of 1678 images. However, we hypothesised that after a certain amount of training data, further increasing the training datasets (TD) yields marginal improvements. Our objective was to show on clinically relevant data that cephalometric landmark predictions of an AI-model facilitate more accurate angle and proportional calculations, thereby enabling a proper orthodontic diagnosis in a shorter time. It presents the novel finding, that given sufficient and high-quality data, an AI-model can serve as a precise diagnostic tool in both spatial and temporal contexts, outlining its advantages and potential drawbacks.

## Materials and methods

The study was approved by the IRB of Semmelweis University, Budapest, Hungary (SE-RKEB number:112/2021). Due to the retrospective nature of the study, the ethics committee waived the requirement for informed consent.

Using four different dataset quantities, we trained a new AI-model developed by Ceph Assistant Ltd. (Budapest, Hungary) and evaluated it on a test dataset. We expected the accuracy and time efficiency of the model to improve as the TD increased, reflecting this progress in our results.

### Data collection

**Figure 1:**
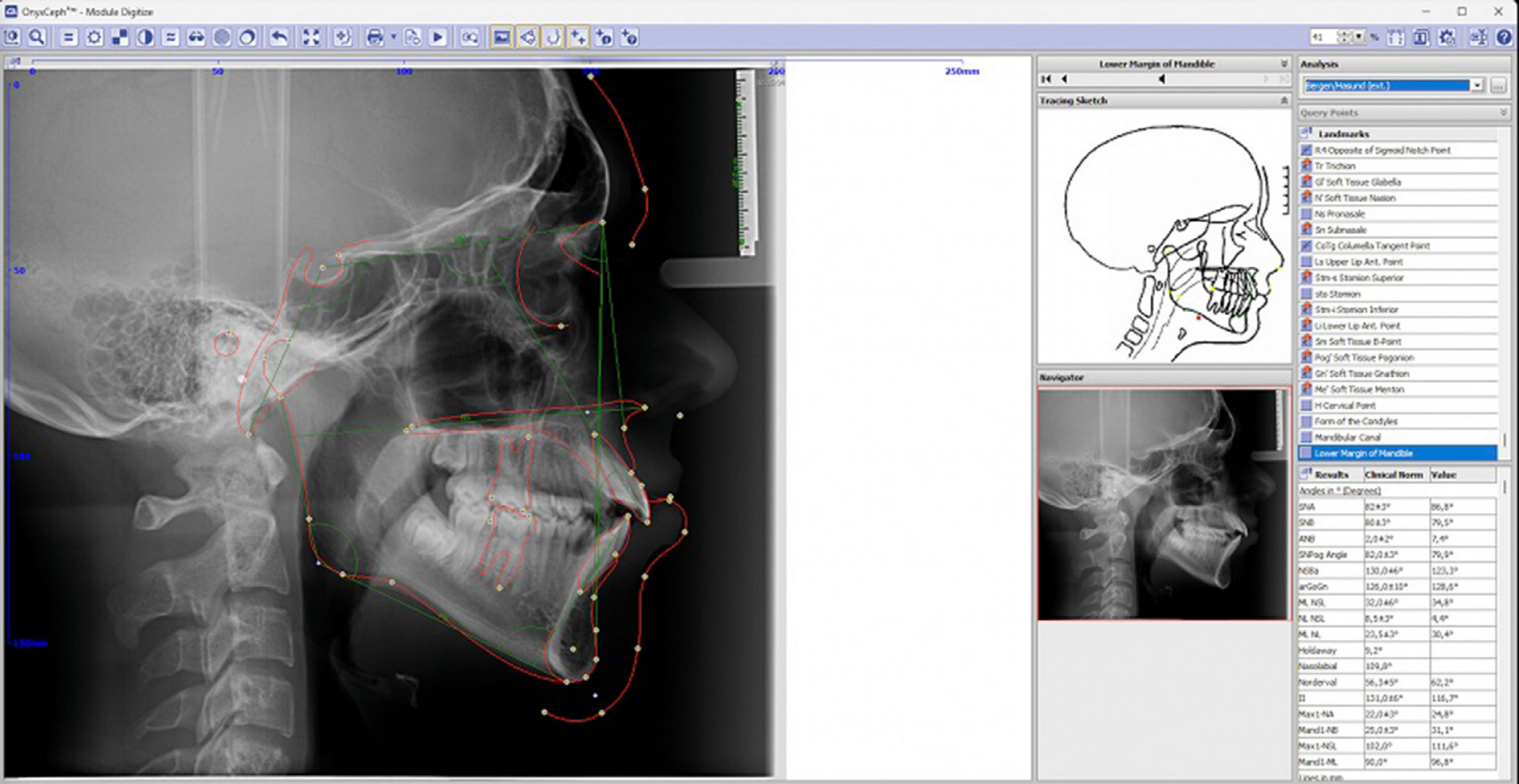
Interface for manual evaluation in the OnyxCeph3TM software.

Regardless of whether the radiographs showed dentures or orthodontic appliances, a total of 1678 2D lateral cephalometric images (2485 × 2232), all uniformly downsampled to a pixel size of 512 × 512, were randomly selected and anonymously downloaded from the OnyxCeph3TM (Chemnitz, Germany) server at Semmelweis University, Department of Paediatric Dentistry and Orthodontics (Budapest, Hungary)^14^ (hereafter‘Clinic’). Altogether, 1600 cephalograms were manually (using mouse-controlled cursor) evaluated by the orthodontists working at the Clinic based on Hasund and Rickett's analysis in the OnyxCeph3TM software (Fig. 1). Calibration and resolution elements of the recordings were checked, and the cephalometric evaluations were verified by three experienced professionals. The X and Y coordinates of each of the 48 landmarks were saved separately, exported and used to train the Ceph Assistant AI-algorithm.^13^

A test dataset consisting of 78 cephalograms was used, randomly selected from a total of 1678 images. To prove the representativeness of the test dataset, cross-validation was performed on a set of 39 (50 %) and 20 (25 %) elements randomly selected 10000 times from the 78 test samples. We calculated the percentage fluctuation of prediction for comparing AI versus AI-corrected and AI versus gold standard as well, to confirm the role of sample selection data over prediction or any reference.

### Training process and technical features of four prediction models with varying training levels

This study utilised the Ceph Assistant^13^ AI-architecture, a Convolutional Neural Network (CNN) specifically developed for landmark localization in lateral cephalometric images, as a reference AI-based cephalometric solution. The TD consisted of a total of 1600 lateral cephalograms, together with their corresponding preliminary manual evaluations stored in .xls/.xlsx format. The model underwent training on four distinct datasets, containing 400/800/1200 and 1600 images, respectively. During each training process, the model received cephalometric images as input and the manually recorded location data of the 48 landmarks as output.

### Testing at four different levels of the model

**Figure 2:**
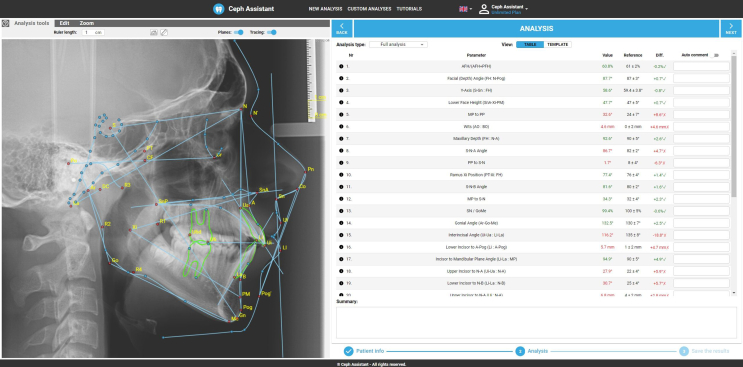
Interface for manual evaluation in the Ceph Assistant software.

During testing, the dataset was automatically analysed by the AI-model following each training set (TD = 400/800/1200/1600 cephalometric images). Once the test set was processed by the AI-algorithm, the senior examiner manually corrected landmark errors using mouse-controlled dot tracing. Manual evaluation of the dataset was performed using a test environment of the Ceph Assistant (Fig. 2), configured directly for this experiment. The evaluation was performed by two experts with 4 (medior) and 10 (senior) years of clinical orthodontic experience. The 78 cephalograms included images of 41 female and 37 male patients with an average age of 13.8 years. Both manual and AI evaluations included 48 cephalometric landmarks of skeletal, dental and profile markers (Table 1). Time was automatically measured by the software.

### Statistical analysis

A statistical analysis was performed on time efficiency and accuracy of landmark detection achieved by the different methods. After measurements were performed ten times (2“manual”, 4“AI-corrected”, 4“AI”), data were compared to the gold standard. The average of the landmark coordinates, corrected by the senior expert on four different occasions, was defined as the gold standard. It was found that the quality of the test images substantially influenced the decision of the experts on landmark positioning and time taken for evaluation. Therefore, experts graded images based on quality and statistical analysis was extended to detect differences. This included precise calibration to accurately compare different images. Table 2 shows that almost a quarter of the images were rated as either easy (score 4, 5) or difficult (score 1, 2) and 42 were rated as moderate (score 3). We employed the violin plot chart to show discrepancies in time measurements, as they can reveal clustering and roughness of distribution, providing additional information.^15^

**Table 2:** Distortion on evaluability of test images by quality scaling with 1–5 scores.

Number of the X-ray	Evaluability score of the X-ray	Gender	Age
1	3	Female	17
2	5	Male	29
3	3	Female	48
4	3	Female	9
5	3	Female	9
6	3	Male	22
7	3	Male	13
8	5	Female	16
9	3	Male	16
10	1	Male	16
11	3	Female	11
12	2	Male	14
13	3	Female	18
14	3	Female	18
15	3	Female	14
16	3	Male	7
17	1	Male	13
18	5	Female	15
19	3	Male	10
20	3	Female	10
21	4	Female	10
22	3	Male	13
23	3	Male	14
24	3	Female	14
25	3	Female	17
26	4	Male	11
27	3	Female	14
28	4	Female	18
29	3	Female	17
30	2	Male	11
31	4	Male	16
32	3	Female	14
33	3	Male	12
34	3	Female	8
35	2	Female	11
36	2	Female	10
37	3	Female	13
38	2	Female	14
39	3	Male	16
40	3	Male	16
41	4	Male	13
42	3	Male	15
43	4	Female	15
44	2	Female	16
45	3	Female	15
46	3	Male	15
47	2	Male	16
48	3	Male	13
49	2	Male	15
50	4	Female	8
51	3	Male	10
52	3	Female	10
53	4	Male	16
54	2	Male	15
55	3	Male	12
56	3	Male	12
57	2	Female	15
58	2	Female	12
59	4	Male	17
60	3	Male	7
61	3	Male	14
62	4	Female	16
63	2	Female	10
64	3	Female	14
65	3	Male	12
66	3	Female	13
67	2	Male	15
68	4	Male	9
69	3	Male	10
70	4	Female	16
71	3	Female	14
72	2	Female	24
73	4	Male	9
74	3	Male	13
75	4	Female	17
76	4	Female	14
77	2	Female	13
78	5	Female	12

The scaling process in this study follows established methodologies for image analysis:

Scaling explanation:

Score 5: Adequately assessed, high-resolution image (total: 2).

Score 4: Adequately assessed, high-resolution image; however, the presence of orthodontic appliances during image acquisition and other factors may have contributed to visible blurry areas, though these are minimally distracting and did not affect analysis integrity (total: 15).

Score 3: Blurred double lines hinder accurate area evaluation, suggesting potential patient movement during image acquisition, complicating analysis (total: 42).

Score 2: Image quality is sub-optimal, with insufficient detail, making it challenging to accurately identify cranial or profile landmarks (total: 17).

Score 1: Image resolution is inadequate, resulting in poor quality and insufficient detail, thereby making it difficult to accurately identify both cranial and profile landmarks (total:2).

Euclidean (L2) distances were considered for distance errors, as L2 performed better than Manhattan (L1) in research where directional information of the coordinates yielded less difference and relevance.^9^^,^^16^ Histograms and a box plot diagram were used to illustrate the comparison between manual versus AI-corrected, manual versus AI-generated and AI-generated versus AI-corrected distances following each TD.

## Results

Although the two references behaved in fundamentally different ways, the percentage fluctuation values were remarkably similar in both the semi-rotation (3.07 %; 3.095 %) and the quarter rotation statistics (5.15 % and 5.29 %). According to these cross-validation values, our test dataset is representative, assuming that the cephalograms cover all relevant clinical cases.

### Time spent on evaluation

**Figure 3:**
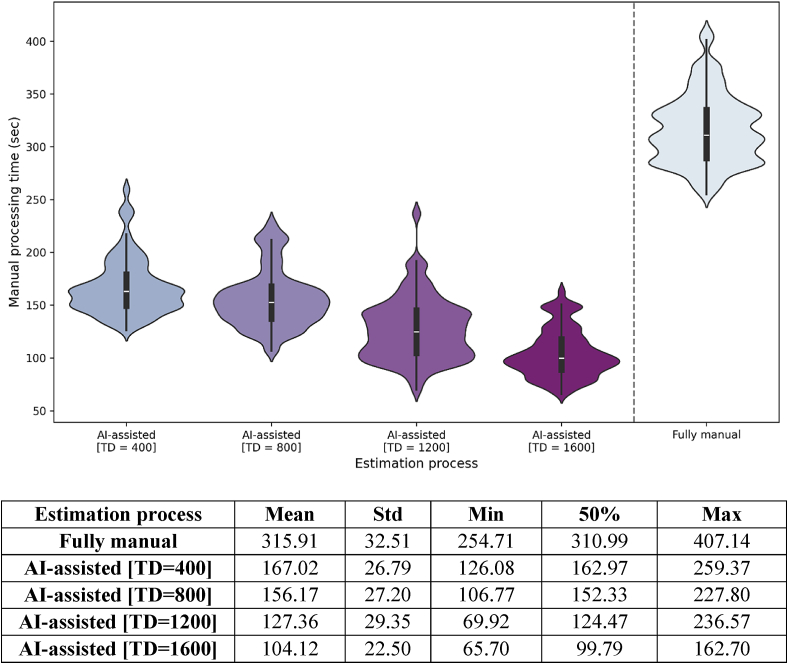
Violin plot diagram on time spent by the two examiners on manual analysis (fully manual) and the time spent by the Ceph Assistant model by varying amounts of training data (TD), on automatic evaluation followed by correction by a single examiner (AI-assisted).

Comparative analysis was performed between the time spent by the two experts on manual cephalometric analysis and the time by the Ceph Assistant model for automatic evaluation, followed by correction of the senior examiner. Manual evaluations required an average of 315.48 s (sec) more than the model predictions (0.43 s) per sample. The first four violin plots in Fig. 3 show that the mean time (104.12–167.02 s) required to correct predictions improved substantially as the AI-algorithm was upgraded with each training set.

### Distance errors of cephalometric landmark tracing methods

We analysed 2D coordinate data (X,Y) of cephalometric landmark predictions (hereafter “AI”) on digital X-ray images after training on all four TDs. We examined the relevance of these predictions to the average of manual corrections of the senior examiner, defined as the gold standard.

**Figure 4:**
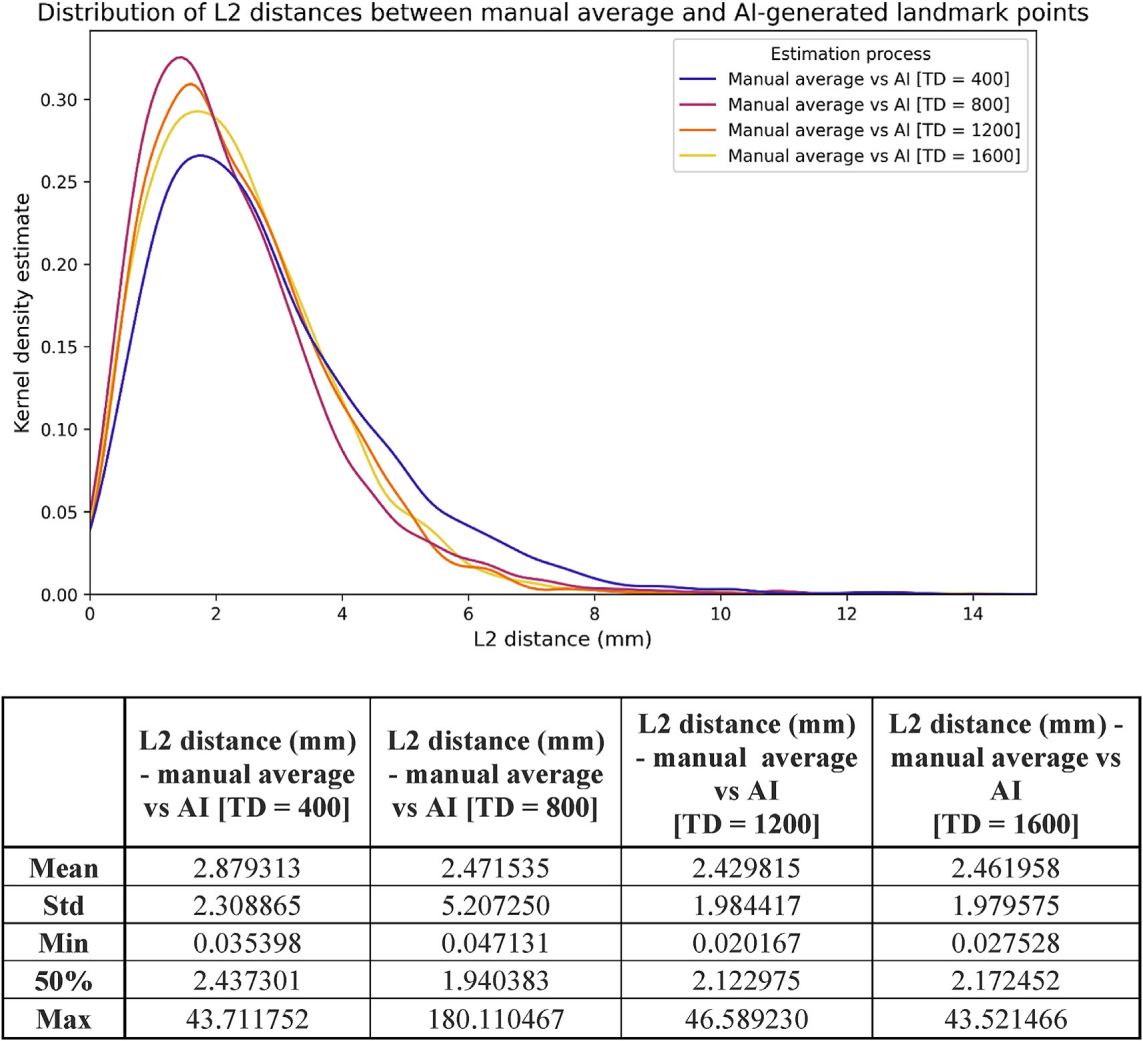
L2 distances between the coordinates of the manual average and AI-generated landmarks by varying amounts of training data (TD).

**Figure 5:**
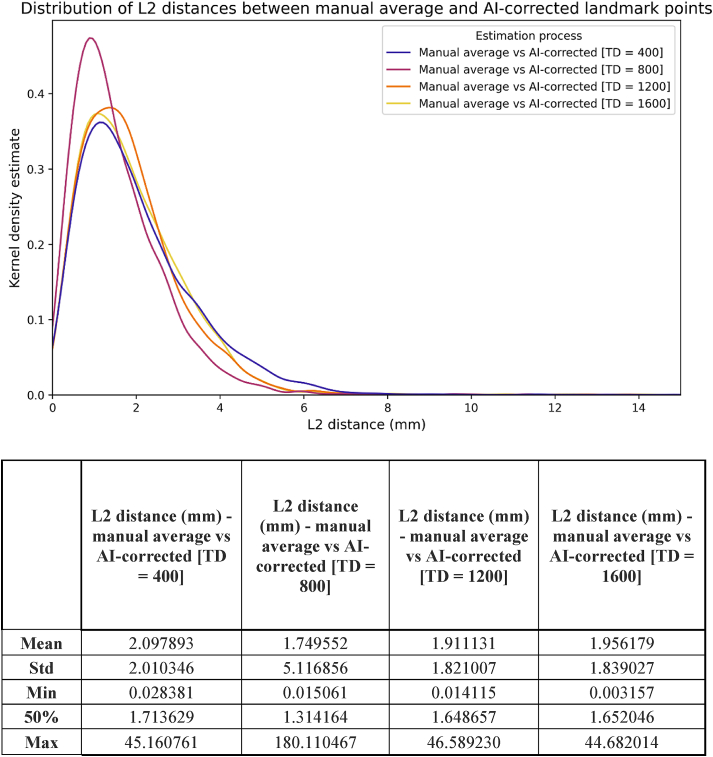
L2 distances between the coordinates of the manual average and AI-corrected landmarks by varying amounts of training data (TD).

Initially, AI was compared to the gold standard (Fig. 4). Mean L2 distances varied between 2.43 and 2.88 mm (median:1.94–2.44 mm) across the four different training levels. Results indicate that the increasing number of training samples (up to TD = 1200) substantially improved the accuracy of the model. Similar results were observed when AI-assisted manual corrections (hereafter “AI-corrected”) were treated as independent variables, with a slightly reduced mean L2 distance of 1.75–2.10 mm (median:1.31–1.71 mm) that remained consistent after TD = 800 (Fig. 5). The noticeable increase in standard deviation after the second measurement was due to variations in decision-making related to the individual circumstances of the examiners in both cases.

**Figure 6:**
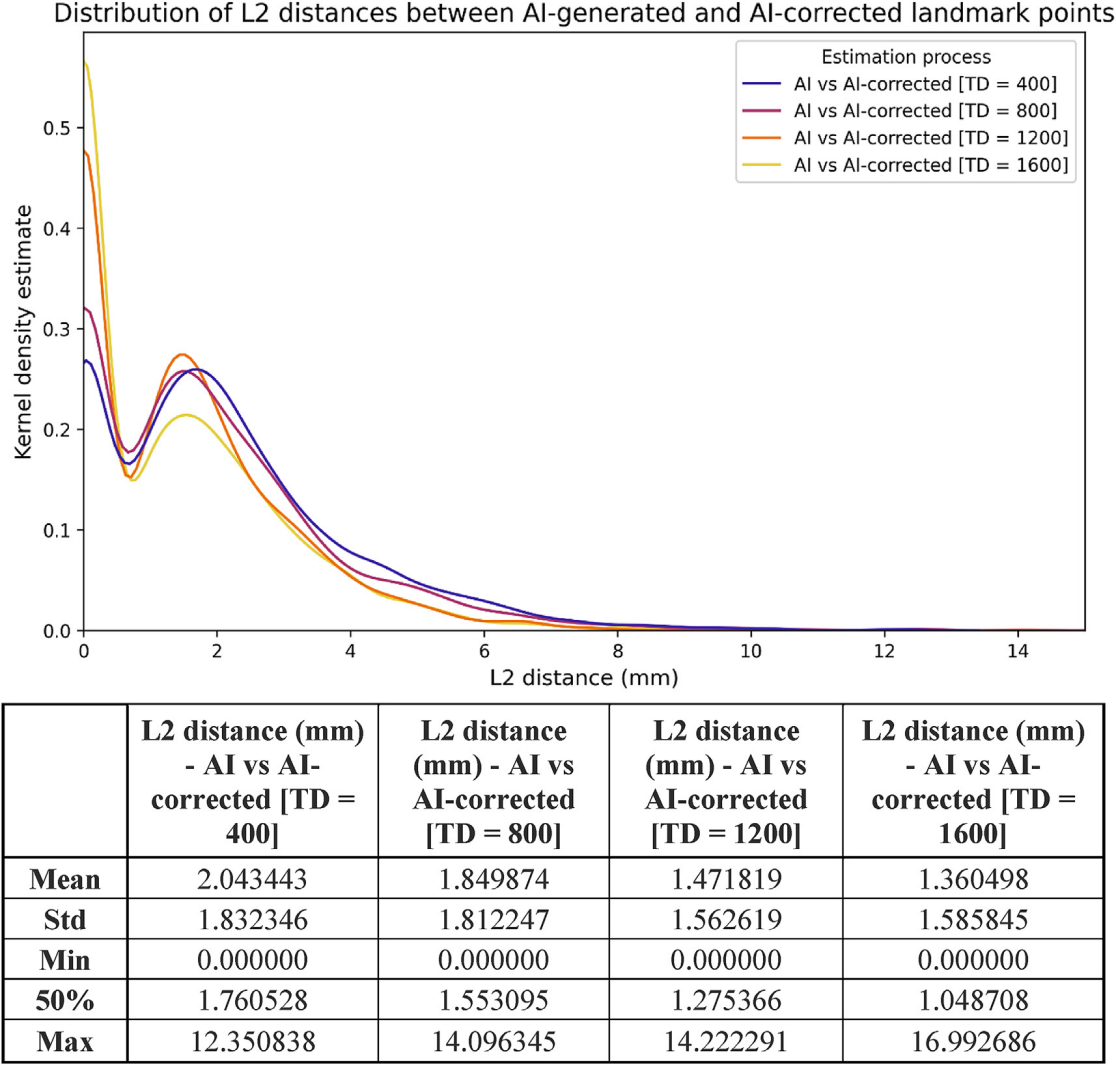
L2 distances between the coordinates of the AI-generated and AI-corrected landmarks by varying amounts of training data (TD).

In addition, we observed substantial agreement between the AI and the AI-corrected landmarks (Fig. 6), with a mean L2 distance ranging from 1.36 to 2.04 mm (median:1.05–1.76 mm), depending on the training level of the model. This highlights the influence of AI on decisions of examiners during manual dot tracing. To eliminate errors due to the subjective bias of a single examiner, a second examiner performed an independent manual evaluation.

**Figure 7:**
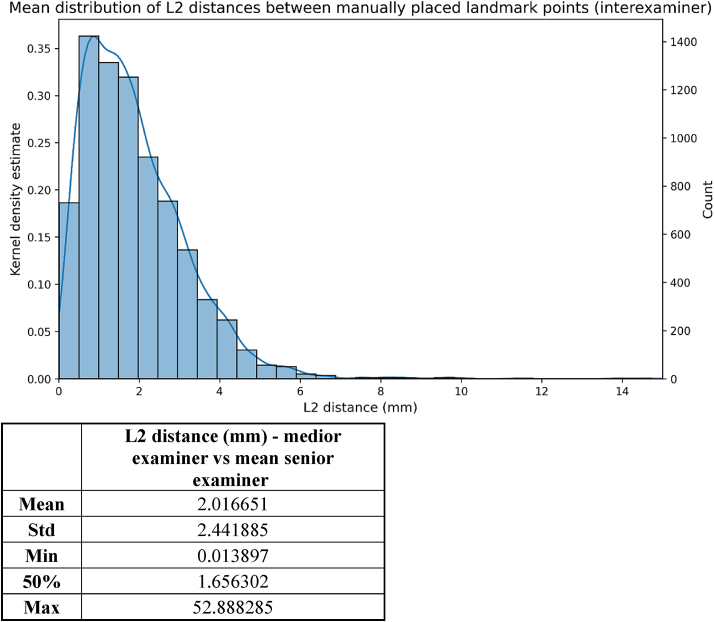
L2 distances between the coordinates of the landmarks detected manually by the two examiners.

**Figure 8:**
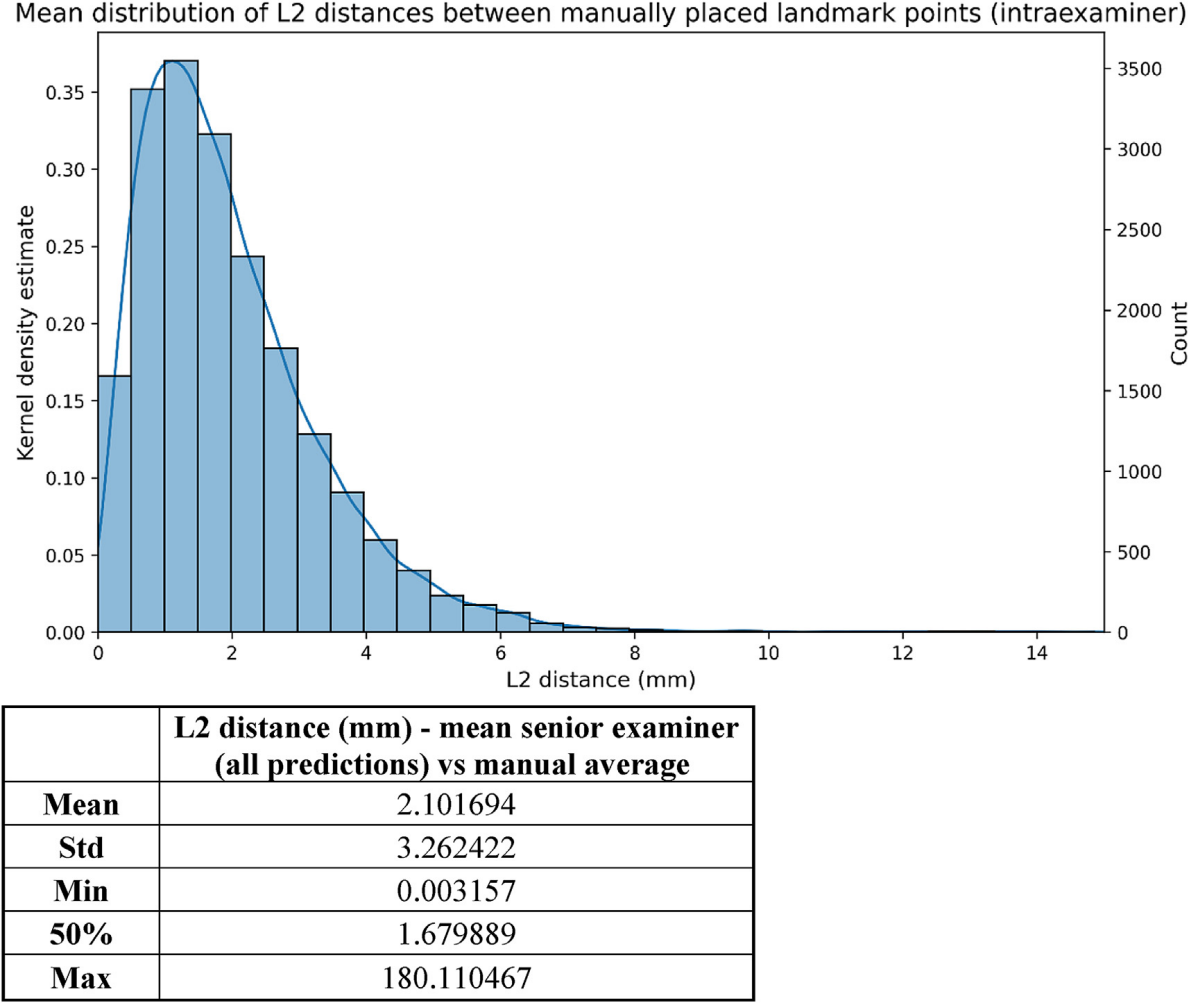
L2 distances between the coordinates of the landmarks detected manually by the senior examiner and the manual average.

**Figure 9:**
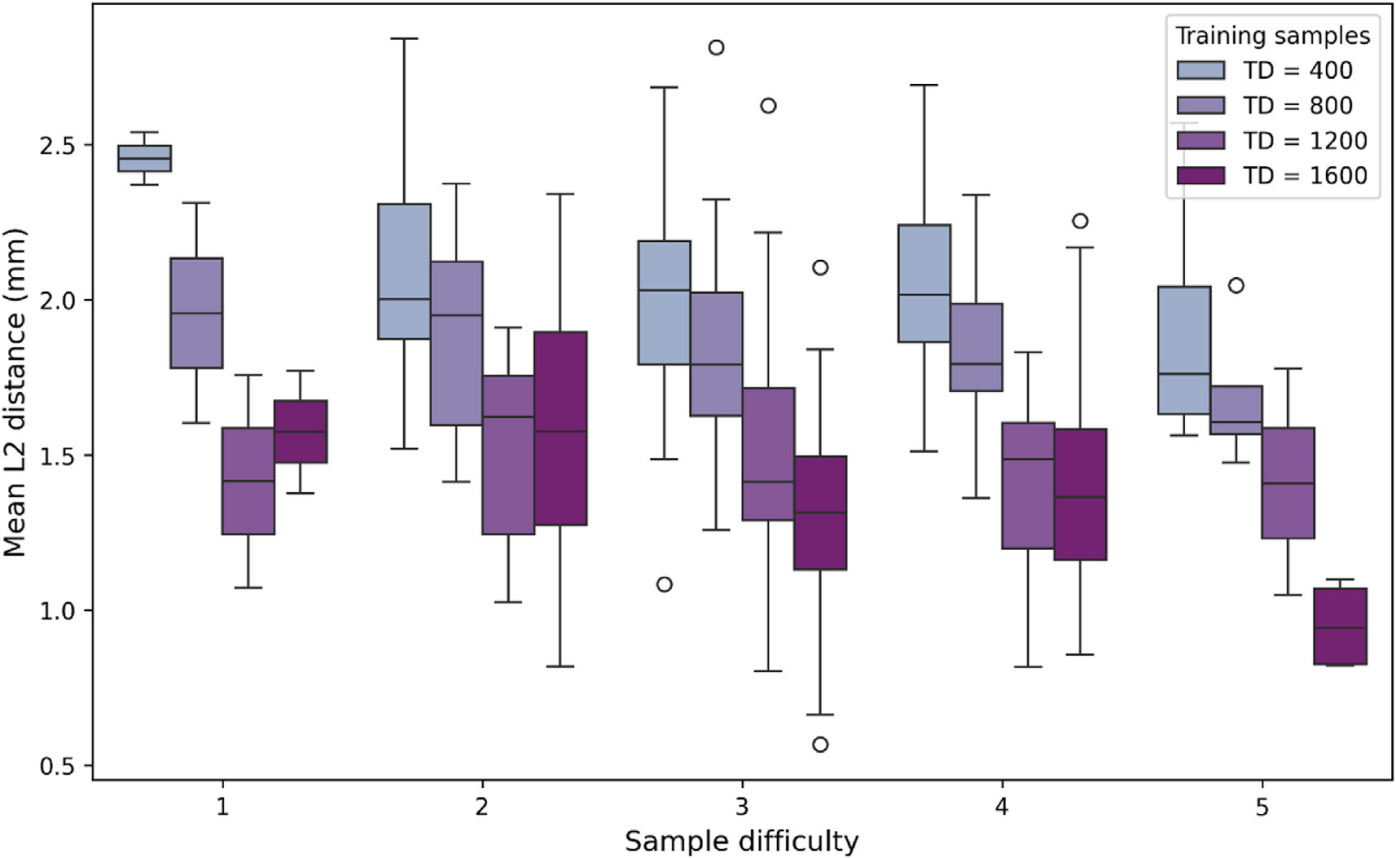
Box plot diagram of the mean L2 distances between the coordinates of the AI-generated and AI-corrected landmarks by varying amounts of training data (TD) and by varying type of image quality.

Comparisons were made between the measurements of the senior and the medior examiners, as well as between the landmarks observed by the senior examiner and the AI-corrected after TD = 1600. The mean L2 distance between the two examiners, representing the inter-examiner error, was 2.02 mm (median:1.66 mm) (Fig. 7), whereas the mean intra-examiner variability was 2.10 mm (median:1.68 mm) (Fig. 8). Furthermore, the results show that initial AI prediction aids the decision making of the clinician, as illustrated by the box plot diagram in Fig. 9. In this case, L2 errors were also evaluated based on the training level of the model and on the complexity of the images (Table 2).

The L2 distance errors of the best models are detailed in Table 3, Table 4. The primary difference between these tables is that in Table 3, model predictions were compared to the gold standard, which is the average of four corrected evaluations by the senior examiner. In contrast, Table 4 shows L2 landmark errors relative to the single correction made after using the latest tool of the model. While the model performs well in comparison to the average, the senior examiner shows larger displacements to correct the prediction than what would be necessary according to the gold standard. On the one hand, this suggests that the actual corrections are smaller than the average. On the other hand, this can be a sign of bias in AI-assistance, as the expert can be influenced by the predictions. However, the placement of fully manual and AI-assisted landmarks is practically acceptable.

**Table 3:** L2 distances between the average of manually corrected model predictions and model predictions after TD = 1600 detailed for each landmark.

	Landmark	Mean L2 distance (mm)	Offset of centers 2D (mm)	Offset of centers X (mm)	Offset of centers Y (mm)
	Cal 1	1.637076	0.232855	0.186644	−0.139232
	Cal 2	1.949541	1.029317	0.701492	−0.753261
1	1LoMma	2.461322	0.592650	−0.343009	0.483300
2	1UpMma	2.464983	1.293880	−1.198248	−0.488188
3	A	2.025425	0.763167	0.401496	0.649018
4	Ar	2.060797	1.300355	−0.527451	1.188579
5	B	2.277940	0.861359	0.654628	0.559823
6	Ba	3.498204	2.640550	−1.940610	1.790680
7	Co	2.398892	0.162771	−0.143024	−0.077709
**8**	**Cond**	**4.031917**	**3.818257**	**3.667022**	**1.063972**
9	D	2.085304	0.680178	−0.614878	0.290805
10	Gl'	2.611720	0.737790	0.138505	0.724672
11	Gn	1.975846	0.435858	−0.207106	0.383510
12	Gn'	2.765901	0.995890	−0.990889	0.099674
13	Id	2.125472	0.570400	0.548000	0.158278
14	Im	3.423167	1.628828	−1.093928	1.206815
15	La	2.399474	1.149321	−0.476898	1.045709
16	Li	2.402736	0.277522	−0.201019	−0.191338
17	Ll	3.064468	1.378565	−0.598300	1.241966
18	Ls1u	2.251858	0.891643	0.102737	−0.885705
19	M6lo	2.493383	1.512762	−1.502171	0.178688
20	Me	2.658894	1.915190	−1.895412	−0.274531
21	Me'	3.249505	2.061361	−2.061004	−0.038344
22	N	1.914186	0.518923	−0.513608	−0.074084
23	N'	2.987097	2.182778	0.270411	−2.165964
24	Or	2.523092	1.193867	−0.569866	1.049081
25	PM	3.278403	1.349037	−0.327414	−1.308702
26	Pn	1.986226	0.270457	0.218972	0.158739
27	Po	2.353079	1.561155	1.072080	−1.134834
28	PoG	1.961738	0.168991	−0.133283	−0.103892
29	Pr	1.916989	0.574963	−0.241903	−0.521599
30	Pt	2.213844	0.713175	−0.602327	−0.381865
31	S	1.441243	0.757301	0.674090	−0.345119
**32**	**Se**	**1.100986**	**0.246422**	**0.152383**	**0.193657**
33	Sm	2.629311	0.842133	0.144136	0.829707
34	Sn	2.268836	0.401509	−0.016119	0.401185
35	SnA	2.260503	0.862765	−0.428025	−0.749105
36	SnP	3.781222	3.401066	−3.319714	−0.739422
37	Stm-i	2.344474	0.736194	0.383469	0.628437
38	Stm-s	2.427442	0.440965	0.342679	−0.277527
39	T1	2.117115	0.371426	0.362880	0.079218
40	T2	3.278450	2.705884	1.354193	2.342642
41	Tr	2.221228	0.636668	−0.449369	−0.451015
42	U6	2.282803	1.167137	−0.850259	−0.799543
43	U6d	3.252854	1.752945	−1.748208	−0.128778
44	Ua	2.062791	0.459479	−0.352060	0.295253
45	Ui	2.201745	0.434141	−0.431062	−0.051614
46	Ul	2.716757	0.864670	−0.263708	−0.823476
47	ppCond	2.767480	2.301227	0.445095	−2.257773
48	sPoG	2.494168	0.809869	−0.179820	−0.789654

Values in bold indicate the most outstanding results.

**Table 4:** L2 distances between model predictions after TD = 1600 and manually corrected model predictions after TD = 1600 detailed for each landmark.

	Landmark	Mean L2 distance (mm)	Offset of centers 2D (mm)	Offset of centers X (mm)	Offset of centers Y (mm)
	Cal 1	0.643981	0.388422	0.384959	−0.051754
	Cal 2	1.236142	1.150663	0.899862	−0.717129
1	1LoMma	2.088942	0.731733	0.065373	0.728807
2	1UpMma	1.780922	0.962528	−0.806648	−0.525146
3	A	0.976627	0.483491	0.299513	0.379546
4	Ar	2.129158	1.561206	−0.423932	1.502546
5	B	1.280191	0.848967	0.792764	0.303761
**6**	**Ba**	**3.410516**	**2.855413**	**−1.792093**	**2.223013**
7	Co	0.387927	0.192925	0.161667	−0.105281
8	Cond	2.922712	2.663729	2.656214	0.199946
9	D	0.462214	0.371593	−0.245463	0.278979
10	Gl'	2.061247	1.395480	0.174731	1.384497
11	Gn	0.560286	0.366428	0.132685	0.341561
12	Gn'	0.731179	0.342332	−0.332368	0.081989
13	Id	1.274109	0.771261	0.764531	0.101660
14	Im	2.939248	1.598290	−0.378901	1.552728
15	La	1.135205	0.917862	−0.213480	0.892691
16	Li	1.555915	0.068109	−0.019415	−0.065283
17	Ll	2.131087	1.321900	−0.435440	1.248124
18	Ls1u	0.840191	0.609058	0.200682	−0.575047
19	M6lo	1.660013	0.942936	−0.890973	0.308700
20	Me	1.422967	1.346214	−1.346151	0.013062
21	Me'	1.287331	1.087110	−1.081382	0.111444
22	N	0.959346	0.356402	−0.308718	−0.178088
23	N'	0.780227	0.679803	−0.108791	−0.671042
24	Or	1.458742	0.975813	−0.348082	0.911619
25	PM	1.051151	0.876947	0.565909	−0.669912
26	Pn	0.413855	0.238079	0.234168	−0.042974
27	Po	1.871023	1.625108	0.684366	−1.473980
28	PoG	0.310770	0.095398	0.086747	−0.039696
29	Pr	0.853830	0.389805	−0.016424	−0.389459
30	Pt	1.779997	0.548484	−0.542283	0.082244
31	S	0.843171	0.659604	0.647199	−0.127323
**32**	**Se**	**0.224547**	**0.026718**	**−0.007611**	**0.025611**
33	Sm	1.767907	0.954243	0.228237	0.926546
34	Sn	0.876506	0.445288	0.313968	0.315762
35	SnA	1.712885	0.765973	−0.270384	−0.716664
36	SnP	2.817688	2.599645	−2.529856	−0.598315
37	Stm-i	0.596301	0.336120	0.037379	0.334035
38	Stm-s	0.544540	0.130432	0.084280	−0.099546
39	T1	1.369595	0.212281	0.211934	0.012134
40	T2	2.321326	1.976671	0.464824	1.921241
41	Tr	0.377330	0.073026	0.040836	0.060541
42	U6	1.407054	0.915504	−0.784272	−0.472297
43	U6d	1.645479	0.923445	−0.759399	0.525418
44	Ua	1.140231	0.643875	−0.631138	−0.127439
45	Ui	1.133775	0.076869	0.018179	0.074689
46	Ul	1.276720	0.878373	0.027640	−0.877938
47	ppCond	2.602839	2.383307	0.052730	−2.382724
48	sPoG	0.969945	0.469860	0.033531	−0.468662

Values in bold indicate the most outstanding results.

When L2 errors were examined for the AI-corrected landmarks after TD = 1600, the highest distances were measured by Condylon (4.03 mm), while the lowest was measured by the center of Sella's entry (1.1 mm) (Table 3). Slightly modified, when the L2 errors were compared between the AI-corrected and the model predicted landmarks after TD = 1600, the highest errors were observed by Basion (3.41 mm), while the lowest errors were found by the center of Sella's entry (0.22 mm) (Table 4).

### Errors in clinically relevant diagnostic values

In orthodontics, diagnostically and therapeutically relevant data (angles, proportions) typically involve at least three landmarks. Therefore, L2 landmark errors of X and Y coordinates provide limited insight into clinical relevance. We performed calculations to assess how L2 discrepancies were reflected in specific orthodontic reference angles or proportions. The mean angular difference between the three landmarks predicted by the model after TD = 1600, and those determined manually ranged from 0.17° to 1.09° on average (Table 5). Similarly, angular difference was valued from 0.05° to 1.86° when angles were determined by four cephalometric landmarks (Table 6). Rational divergence was observed in the proportion of lower and upper facial heights, determined by three landmarks (N, SnA, Me) with the prediction showing a difference of 3.14 % from the gold standard ratio after TD = 1600 were completed on the algorithm.

**Table 5:** Angular differences between model prediction after TD = 1600 and manual average (angles determined by three cephalometric landmarks).

Reference	Method	Mean reference angle (deg)	Mean predicted angle (deg)	Mean angular difference (deg)
SNA angle	Manual average and AI 1600	−82.087991	−80.997427	**1.090385**
SNB angle	Manual average and AI 1600	−77.652970	−76.731267	**0.922000**
ANB angle	Manual average and AI 1600	4.435581	4.267428	**−0.168466**
SNPog angle	Manual average and AI 1600	−78.659431	−78.349143	**0.310633**

**Table 6:** Angular differences between model prediction after TD = 1600 and manual average (angles determined by four cephalometric landmarks).

Reference	Method	Mean reference angle (deg)	Mean predicted angle (deg)	Mean angular difference (deg)
Facial angle	Manual average and AI 1600	90.729613	92.592710	1.861190
Gonion angle	Manual average and AI 1600	120.646687	119.763093	−0.886658
Interincisal angle	Manual average and AI 1600	128.086133	126.961123	−1.117114
IMPA angle	Manual average and AI 1600	98.796140	98.769588	−0.053833

## Discussion

We maintained high-quality training and evaluation data by following standardized protocols and consistent measurement procedures while including samples with diverse medical and imaging characteristics to ensure comprehensive domain coverage. Additionally, we conducted thorough statistical analyses using medically relevant metrics and perspectives to support the development of a reliable AI model.

In terms of time efficiency, modern prediction tools are advantageous for cephalometric evaluation. Even with corrections of landmarks predicted by models trained on smaller datasets, the evaluation took less than half the time of fully manual dot tracing. In terms of accuracy, the mean L2 distance error of AI after TD = 1600 was 66 % of the difference between the manual tracing of the two experts using the same metric, showing that the latest model provided more consistent dot tracing than two different examiners or the same examiner on two different occasions. These results confirm the hypothesis that examiners make minor corrections to AI, indicating that predictions influence the decisions of the examiner during cephalometric analysis; however, these potentially biased placements are still medically correct, and were even closer to the gold standard, indicating that model assistance may not only speed up but also improve manual prediction. The extension of TD improved model precision, but these small improvements are clinically insignificant, as the tool noise is lower than examiner noise. Still, the use of higher quality models is beneficial as they yield considerably better results with less correction. Previous studies suggest lower intra- and inter-examiner variability compared to our findings, which may be due to variations in exclusion criteria and the increased number of landmarks incorporated in our study.^17^^,^^18^

According to Wang et al.,^19^, ^20^, ^21^ landmark detection within 2 mms is clinically acceptable. Although our model slightly exceeded this criterion on average, the median prediction distance is well inside this threshold, probably due to the fact that our evaluation scheme included much more difficult landmarks than many other studies. Furthermore, our calculations of clinically relevant angular and ratio errors between AI and human dot tracing showed promising results.

Considering these references, we can claim that our best prediction model can serve as an accurate baseline for orthodontic analysis on lateral cephalograms, substantially speeding up the workflow of orthodontic diagnostics.^22^ Given the large amount and high quality of data available through this method, a fully autonomous system could be developed that requires no corrections. Further research could investigate the consequences of evaluation errors and biases in clinical therapy. Future research should focus on training AI with malocclusion-specific datasets and integrating diverse evaluation methods to create a robust, precise, and efficient AI-driven diagnostic system for clinical practice.

## Declaration of Generative AI and AI-assisted technologies in the writing process

During the preparation of this article the authors used ChatGPT in order to improve the readability and language of the paper. After using this Generative AI-tool/service on certain sentences and expressions, the authors reviewed and edited the content as needed and take full responsibility for the content of the publication.

## Declaration of competing interest

The authors have no conflicts of interest relevant to this article.

## References

[1] Xie B., Xu D., Zou X.Q., Lu M.J., Peng X.L., Wen X.J. (2023). Artificial intelligence in dentistry: a bibliometric analysis from 2000 to 2023. J Dent Sci.

[2] Khanagar S.B., Al-Ehaideb A., Maganur P.C. (2021). Developments, application, and performance of artificial intelligence in dentistry - a systematic review. J Dent Sci.

[3] Khanagar S.B., Al-Ehaideb A., Vishwanathaiah S. (2021). Scope and performance of artificial intelligence technology in orthodontic diagnosis, treatment planning, and clinical decision-making - a systematic review. J Dent Sci.

[4] Lee H.T., Chiu P.Y., Yen C.W., Chou S.T., Tseng Y.C. (2024). Application of artificial intelligence in lateral cephalometric analysis. J Dent Sci.

[5] Kim J., Kim I., Kim Y.J. (2021). Accuracy of automated identification of lateral cephalometric landmarks using cascade convolutional neural networks on lateral cephalograms from nationwide multi-centres. Orthod Craniofac Res.

[6] Park J.H., Hwang H.W., Moon J.H. (2019). Automated identification of cephalometric landmarks: Part 1-Comparisons between the latest deep-learning methods YOLOV3 and SSD. Angle Orthod.

[7] Kunz F.A.-O., Stellzig-Eisenhauer A., Zeman F., Boldt J. (2020). Artificial intelligence in orthodontics: evaluation of a fully automated cephalometric analysis using a customized convolutional neural network. J Orofac Orthop.

[8] Kang S., Kim I., Kim Y.J., Kim N., Baek S.H., Sung S.J. (2024). Accuracy and clinical validity of automated cephalometric analysis using convolutional neural networks. Orthod Craniofac Res.

[9] Ye H., Cheng Z., Ungvijanpunya N., Chen W., Cao L., Gou Y. (2023). Is automatic cephalometric software using artificial intelligence better than orthodontist experts in landmark identification?. BMC Oral Health.

[10] Durão A.R., Pittayapat P., Rockenbach M.I. (2013). Validity of 2D lateral cephalometry in orthodontics: a systematic review. Prog Orthod.

[11] Mahto R.K., Kafle D., Giri A., Luintel S., Karki A. (2022). Evaluation of fully automated cephalometric measurements obtained from web-based artificial intelligence driven platform. BMC Oral Health.

[12] Meriç P., Naoumova J. (2020). Web-based fully automated cephalometric analysis: comparisons between App-aided, computerized, and manual tracings. Turkish J Orthod.

[13] Webpage of Ceph Assistant Ltd., Budapest, Hungary. https://www.cephassistant.com/.

[14] Webpage of Semmelweis University, Department of Paediatric Dentistry and Orthodontics, Budapest, Hungary. https://semmelweis.hu/gyermekfogaszat/english/.

[15] Hintze J.L., Nelson R.D. (1998). Violin plots: a box plot-density trace synergism. Am Statistician.

[16] Malkauthekar M. (2013). Third International Conference on Computational Intelligence and Information Technology (CIIT 2013), Mumbai, India.

[17] Hwang H.W., Park J.H., Moon J.H. (2020). Automated identification of cephalometric landmarks: Part 2-Might it be better than human?. Angle Orthod.

[18] Arık S.Ö., Ibragimov B., Xing L. (2017). Fully automated quantitative cephalometry using convolutional neural networks. J Med Imaging.

[19] Wang C.W., Huang C.T., Hsieh M.C. (2015). Evaluation and comparison of anatomical landmark detection methods for cephalometric X-ray images: a grand challenge. IEEE Trans Med Imag.

[20] Wang C.W., Huang C.T., Lee J.H. (2016). A benchmark for comparison of dental radiography analysis algorithms. Med Image Anal.

[21] Lindner C., Wang C.W., Huang C.T., Li C.H., Chang S.W., Cootes T.F. (2016). Fully automatic system for accurate localisation and analysis of cephalometric landmarks in lateral cephalograms. Sci Rep.

[22] Chen Y.J., Chen S.K., Yao J.C., Chang H.F. (2004). The effects of differences in landmark identification on the cephalometric measurements in traditional versus digitized cephalometry. Angle Orthod.

